# Blast transformation and fibrotic progression in polycythemia vera and essential thrombocythemia: a literature review of incidence and risk factors

**DOI:** 10.1038/bcj.2015.95

**Published:** 2015-11-13

**Authors:** S Cerquozzi, A Tefferi

**Affiliations:** 1 Division of Hematology, Department of Medicine, Mayo Clinic, Rochester, MN, USA

## Abstract

Polycythemia vera (PV) and essential thrombocythemia (ET) constitute two of the three *BCR-ABL1*-negative myeloproliferative neoplasms and are characterized by relatively long median survivals (approximately 14 and 20 years, respectively). Potentially fatal disease complications in PV and ET include disease transformation into myelofibrosis (MF) or acute myeloid leukemia (AML). The range of reported frequencies for post-PV MF were 4.9–6% at 10 years and 6–14% at 15 years and for post-ET MF were 0.8–4.9% at 10 years and 4–11% at 15 years. The corresponding figures for post-PV AML were 2.3–14.4% at 10 years and 5.5–18.7% at 15 years and for post-ET AML were 0.7–3% at 10 years and 2.1–5.3% at 15 years. Risk factors cited for post-PV MF include advanced age, leukocytosis, reticulin fibrosis, splenomegaly and *JAK2V617F* allele burden and for post-ET MF include advanced age, leukocytosis, anemia, reticulin fibrosis, absence of *JAK2V617F*, use of anagrelide and presence of *ASXL1* mutation. Risk factors for post-PV AML include advanced age, leukocytosis, reticulin fibrosis, splenomegaly, abnormal karyotype, *TP53* or *RUNX1* mutations as well as use of pipobroman, radiophosphorus (P^32^) and busulfan and for post-ET AML include advanced age, leukocytosis, anemia, extreme thrombocytosis, thrombosis, reticulin fibrosis, *TP53* or *RUNX1* mutations. It is important to note that some of the aforementioned incidence figures and risk factor determinations are probably inaccurate and at times conflicting because of the retrospective nature of studies and the inadvertent labeling, in some studies, of patients with prefibrotic primary MF or ‘masked' PV, as ET. Ultimately, transformation of MPN leads to poor outcomes and management remains challenging. Further understanding of the molecular events leading to disease transformation is being investigated.

## Introduction

Myeloproliferative neoplasms (MPN) are hematopoietic stem cell malignancies characterized by clonal proliferation of myeloid-lineage cells. The four popular (also known as ‘classic') MPN are chronic myeloid leukemia, polycythemia vera (PV), essential thrombocythemia (ET) and primary myelofibrosis (PMF).^1^ PV, ET and PMF are operationally referred to as *BCR-ABL1*-negative MPN and are characterized by recurrent *JAK2*, calreticulin (*CALR*) or myeloproliferative leukemia virus oncogene (*MPL*) mutations.

In a recently published study with mature survival data, life-expectancy was significantly compromised in all three *BCR-ABL1*-negative MPN and median survivals for PV and ET were reported at approximately 14 and 20 years, respectively, and in those <60 years of age at 24 and 30 years.^2^ The International Working Group for MPN Research and Treatment (IWG-MRT) have identified thrombosis history, leukocytosis and advanced age as independent risk factors for overall survival in both PV and ET (Table 1).^3, 4^ Disease-related complications affecting survival in both PV and ET include thrombohemorrhagic events and disease transformation into myelofibrosis (MF) or acute leukemia, also known as ‘blast phase (BP)' disease. The latter is often in the form of acute myeloid leukemia (AML) although cases of lymphoblastic transformation have been reported.^5, 6, 7^ Post-MPN AML has dismal prognosis with median survival of <6 months and long-term remissions can only be achieved through allogeneic stem cell transplant.^8, 9^ Here we review the reported rates of both fibrotic and leukemic transformation (LT) in PV and ET and the risk factors associated with disease progression.

## Post-polycythemia vera myelofibrosis (post-PV MF)

Post-PV MF represents a natural evolution of PV and is defined based on IWG-MRT consensus criteria requiring a history of a World Health Organization (WHO)-diagnosed PV and bone marrow fibrosis grade ⩾2 (3-point scale) or ⩾3 (4-point scale). At least two of the following features must also be present: anemia or sustained loss of need for phlebotomy and/or cytoreductive therapy, a leukoerythroblastic peripheral smear, splenomegaly and one or more constitutional symptoms.^10^ Less than 10% of PV patients evolve into MF within their first decade with reported incidences shown in Table 2 ranging from 2.3% to 23%.^2, 3, 7, 11, 12, 13, 14, 15, 16, 17, 18, 19, 20^ The cumulative incidence of MF evolution is 5–14% at 15 years.^7, 11, 17^ Interestingly, a recent cohort of Chinese PV patients demonstrated significantly higher 10-, 15- and 20-year incidences of post-PV MF at 27.4, 39.9 and 61.1%, which may suggest that this population of patients are at higher risk of fibrotic transformation.^20^ Ultimately, transformation to MF shortens PV survival rates (hazards ratio (HR)=2.17; confidence interval (CI): 1.27–3.72, *P=*0.005), after adjusting for prognostic factors such as age, white blood cell count, hemoglobin level, platelet count and spleen size.^21^ Older age (⩾60 years) and leukocytosis (>10 or 15 × 10^9^/l) increases the risk for post-PV MF evolution.^14, 21, 22^ Based on both retrospective and prospective studies, median time to MF transformation is 8.5–20 years from time of diagnosis.^2, 7, 11, 14, 17, 18, 19^ PV patients ⩽45 years have a longer median time to MF transformation of 20 years compared with transforming in a median 8 years for patients who are ⩾65 years.^18^ In a large prospective multicentered cohort of 1638 PV patients, 38 (2%) transformed to MF whereby greater duration of disease impacted transformation rates. PV patients with a disease course of 6–10 years had a relative risk of fibrotic transformation of 5.74; 95% CI, 1.51–21.77 and while disease duration >10 years resulted in relative risk of 15.24; 95% CI, 4.22–55.06; *P<*0.0001.^13^ Additional risk factors for post-PV MF include: presence of baseline bone marrow fibrosis, *JAK2V617* allele burden, splenomegaly, thrombocytosis (platelet count >550 × 10^9^/l) and the presence of a ‘masked PV' phenotype (display PV-characteristic BM morphology but lower hemoglobin levels than WHO criteria targets).^15, 16, 20, 23, 24, 25^ Once transformed to post-PV MF, median survival is drastically shortened to 5.7 years.^21^ Passamonti *et al.*^21^ developed a dynamic prognostic model for patients who developed post-PV MF based on three independent risk factors: hemoglobin <100 g/l, platelet count <100 × 10^9^/l, and leukocyte count >30 × 10^9^/l. The presence of any of these risk factors results in a 4.2-fold increased risk of death. Anemia, at the time of MF transformation, leads to significant differences in survival of 6.6 versus 1.9 years for patients with hemoglobin ⩾100 g/l compared with <100 g/l, respectively (*P<*0.001).^21^ Similarly, among a Chinese cohort, anemia (hemoglobin <100 g/l) and age >65 years significantly predicted worse outcomes, resulting in a 5-year survival rate of only 17.3% (median 3 years) in post-PV MF patients with both risk factors.^20^

## Post-polycythemia vera acute myeloid leukemia

Based on large multicenter PV patient data, rates of LT in PV are estimated at 2.3% at 10 years, 5.5% at 15 years and remain <10% at 20 years.^2, 3^ Higher cumulative incidence rates or actuarial risks of LT of 8–14% at 10 years, 14–19% at 15 years and up to 24% at 18 years have been reported in smaller studies.^7, 17^ As outlined in Table 2, LT typically occurs within a median time of 4.6–19 years from initial PV diagnosis.^2, 3, 7, 11, 12, 14, 17, 18, 19^ Younger patients (⩽45 years) transform to leukemia at a median time of 19 years compared with 7 years for patients aged >65 years, but the difference is not statistically significant (*P=*0.37). Rates of both MF and LT transformation occur at the same frequency between age groups (15% versus 10%, *P=*0.29), although, as expected, leukemic development contributed to more deaths in older patients.^18^ Factors influencing leukemic-free survival included: age (>61 years^3^ or ⩾70 years),^12, 13^ leukocytosis (⩾10 × 10^9^/l or ⩾15 × 10^9^/l),^3, 7, 14^ abnormal karyotype,^3^ splenomegaly, and bone marrow reticulin grade.^16^ In the two largest prospective studies including >1500 PV patients, age >61 years (HR 6.3; 95% CI, 1.2–13.1, *P=*0.03)^3^ or >70 years (HR 4.30; 95% CI, 1.16–15.94, *P=*0.0294)^12^ and leukocytosis ⩾15 × 10^9^/l (HR of 3.9; 95% CI, 1.3–11.6, *P=*0.0004) adversely impacted LT risk.^3^ Female sex was a risk factor in a single study by Finazzi *et al.*^12^ Lower cholesterol levels (⩽150 mg/dl) has been linked to AML/myelodysplastic syndrome (MDS) transformation (HR 6.58; 95% CI, 2.08–20.86, *P=*0.0014) and is seen in advanced stages of proliferative disease possibly representing a marker of disease activity.^12, 26^ Multiple treatment regimens have also been linked to leukemic risk and will be discussed in greater detail below. Multivariate analysis has shown association of alkylating agents (pipobroman, P^32^, chlorambucil) and leukemia.^3, 11, 12, 13^ In particular, exposure to P^32^, busulphan and pipobroman either alone or in combination has been linked to higher LT (HR of 5.46; 95% CI, 1.84–16.25, *P=*0.0023), with no LT risk associated with hydroxyurea (HU) usage alone (HR 0.86, 95% CI, 0.26–2.88, *P=*0.8021).^12^ HU remains a standard cytoreduction therapy in PV, and it is important to recognize that HU resistance increases both LT and MF transformation risk (HR 6.8; 95% CI, 3.0–15.4%, *P<*0.001), whereas HU intolerance has no direct effect on outcomes.^27^

## Post-essential thrombocythemia myelofibrosis (post-ET MF)

Post-ET MF is defined based on IWG-MRT consensus criteria requiring a previously documented WHO diagnosis of ET and the presence of bone marrow fibrosis (grade ⩾2 on a 3-point scale or ⩾3 on a 4-point scale). Two or more additional features are needed, including: anemia (with a ⩾2 mg/l decrease from baseline), leukoerythroblastic peripheral blood findings, splenomegaly, elevated lactate dehydrogenase and ⩾1 constitutional symptoms.^10^ Transformation to MF is less frequent in ET than PV and occurs late during the course of the disease.^11, 22, 28^ In larger cohort studies, the cumulative risk of post-ET MF is 3.9% at 10 years (incidence of 3.7 × 1000 person-years) and as high as 9.3% at 15 years. Table 3 summarizes rates of transformation among ET patients. Cumulative probabilities of fibrotic progression have been reported as 19.9% (5.6–12.6%) at 20 years.^28^ Overall, the median time to myelofibrotic transformation is approximately 7–16 years from time of ET diagnosis.^11, 28, 29, 30, 31, 32, 33^ The variation of cumulative incidence rates of transformation in ET are attributed to discrepancies in diagnosis, in particular, distinguishing between primary ET and prefibrotic MF. Barbui *et al.*^34^ compared the outcomes between confirmed ET and prefibrotic MF patients and identified that progression to overt MF at 10 and 15 years was 0.8% and 9.3%, compared with 12.3% and 16.9%, respectively. This correlates to a lower incidence of transformation of 0.5 per 100 patient-year in ET.^34^ Risk factors for MF transformation include: age,^34^ anemia,^16, 22, 30, 34^ and bone marrow histopathology, including hypercellularity and increased reticulin.^16, 33, 34^ The influence of molecular markers such as *JAK2V617F* will be discussed in detail below. Additional studies have found that increased serum lactate dehydrogenase, increased leukocytosis and male gender are risks for myelofibrotic transformation.^16, 30, 33^

## Post-essential thrombocythemia acute myeloid leukemia

Earlier cohort studies of ET patients indicate that LT occurs at an incidence of 3 × 1000 person-years, with a cumulative risk of 2.6% at 10 years and 5.3% at 15 years.^30^ Higher rates of transformation have been found in French, Spanish and Chinese studies, with 10-year rates of 8.3–9.7%.^29, 35, 36^ Conversely, much lower rates of LT have been identified as <1% at 10 years and 2% in 15 years.^11, 34^ Again, this variability in findings has largely been attributed to discrepancies in morphological diagnosis between ET and prefibrotic PMF related to modifications in the 2001 WHO classification of ET to prefibrotic MF. Accordingly, Barbui *et al.*^34^ confirmed ET in 891 patients based on the revised WHO criteria and found lower LT risks of 0.7% at 10 years and 2.1% at 15 years compared with 5.8% and 11.7% in the setting of early/prefibrotic PMF. In that particular study, prefibrotic PMF morphology, previous thrombosis and thrombocytosis (>1000 × 10^9^/l) were identified as risk factors.^34^ Other risk factors for LT include: leukocytosis (⩾15 × 10^9^/l),^16, 29^ extreme thrombocytosis (⩾1000 × 10^9^/l),^34, 37^ anemia,^16, 22, 37^ older age (⩾60 years),^30, 37^ reticulin grading, and bone marrow cellularity.^16, 34^ Among the Mayo clinic cohort of 605 ET patients, anemia (Hgb<120 g/l in females, <135 g/l in males) and thrombocytosis (⩾1000 × 10^9^/l) were found to be significant risk factors, and when incorporated into a prognostic model, patients with no risk factors had a 0.4% versus 6.5% (2 risk factors) rate of LT (*P=*0.0009).^37^

## Therapy-related risks of disease transformation

With the introduction of radiation treatment, in 1965, Modan and Lilienfeld observed that rates of PV-related LT were higher among X-ray-treated (8.9%) and P^32^-treated (11%) patients than the non-radiated (1%) treatment groups.^38^ Both the Polycythemia Vera Study Group (PVSG) and the French Polycythemia Study Group (FPSG) have also illustrated the leukemogenic potential of P^32^, with an incidence of 5–15% after 10 years of observation.^38, 39, 40^ In a large nested case–control study of MPN, AML/MDS development was significantly associated with P^32^ and alkylator exposure.^41^ Among alkylators, pipobroman has leukemogenic potential with actuarial leukemic risk of 14.4% and 18.7% at 10 and 15 years, respectively, reported among 164 PV patients.^7^ When investigating the role of low-dose aspirin in PV patients, the use of P^32^, pipobroman and busulphan independently had an impact on progression to AML/MDS in addition to the combination of either alkylating agent or P^32^ with HU; however, the use of HU alone was not found to be leukemogenic (HR 0.86; 95% CI, 0.26–2.88, *P=*0.8021).^12^ The FPSG published their final results after a median follow-up of 16.3 years randomizing young PV patients (<65 years of age) to either first-line pipobroman or HU. In that study, the median survival was 17 years for the entire cohort compared with 20.3 years for those treated with HU and 15.4 years if received pipobroman (*P=*0.008) and differed significantly from age- and sex-matched general population. The cumulative incidence of LT was 6.6, 16.5 and 24% in the HU arm versus 13, 34 and 52% in the pipobroman arm (*P=*0.004) at 10, 15 and 20 years. This is despite patients being treated for longer durations in the HU arm (12 years) compared with pipobroman (9.5 years, *P<*0.01). When comparing single-agent therapy only (HU *n=*94, pipobroman *n=*130), the cumulative incidence of LT was 7.3, 10.7 and 16.6% compared with 14.6, 34 and 49.4% at 10, 15 and 20 years, respectively (*P=*0.002).^42^ This illustrates not only the leukemogenicity of pipobroman but also identifies the higher rates of LT with HU than previously reported in PV, which may or may not reflect the rate of natural evolution of the disease. In a large cohort of >1500 PV patients, multivariate analysis confirmed the association between LT and P^32^/chlorambucil, pipobroman and pipobroman+HU/busulphan use. The use of HU or busulphan alone or in combination was not associated with leukemic risk.^3^ Likewise, cytoreductive agents unlike HU or interferon-alpha were associated with leukemic risk among the prospective multicenter PV study of 1638 patients.^13^ Although, HU treatment is not leukemogenic, therapy with ⩾2 cytoreductive treatments carries an increased risk of LT (odds ratio (OR) 2.9; 95% CI, 1.4–5.9).^41^ It is important to consider the risks of additional therapy in the rare setting of HU intolerance or refractoriness when deciding on choices for alternative cytoreductive agents.

In contrast, the cumulative incidence of post-PV MF increases with HU at 15, 24 and 32% compared with 5, 10 and 21% using pipobroman (*P=*0.02) at 10, 15, and 20 years, respectively.^42^ Accordingly, sequential use of two or more myelosuppressive agents leads to higher incidences of MF compared with pipobroman use alone.^11^ Although, PV patients receiving myelosuppressive agents had significantly higher MF transformation (*P=*0.01), they also had a significantly longer follow-up period compared with those treated with phlebotomy alone (*P<*0.001) and its was shown that longer follow-up also significantly led to higher rates of post-PV MF, thus making it difficult to conclude the effects of therapy on transformation.^21^

Few studies are available evaluating the efficacy and safety of cytoreductive agents in ET. Palandri *et al.*^31^ conducted a single-institution retrospective study on 386 ET patients with a median follow-up of 9.5 years (3–28.5 years) whereby 88% of the population received cytoreductive therapy. The evolution to AML and MF was 1.5% and 5%, respectively, and transformation was not influenced by the type of cytoreductive therapy: HU or busulphan. However, patients receiving sequential therapy were at higher risk for AML/MDS compared with single-therapy use (*P=*0.0039), and no disease transformation occurred among patients without treatment or if exposed to interferon alone.^31^ Anagrelide has been linked to post-ET MF, when compared with HU in the PT-1 trial and used in combination with aspirin (OR 2.92, 95% CI, 1.24–6.86, *P<*0.01).^43^ Duration of anagrelide use (>60 months) posed a higher risk for transformation (OR 9.32; 95% CI, 1.1–78.5, *P<*0.01)^44^ However, no differences were found in the ANAHYDRET study that evaluated WHO–ET diagnosed patients compared with the UK-PT1 study, which based ET on PVSG criteria.^45^ Finally, melphalan exposure has been associated with increased risk of evolution to AML among ET patients although it is not a commonly used therapy.^29^

Overall, there is no confirmed consensus implicating HU, anagrelide or busulphan as leukemogenic agents in PV or ET. The two largest non-controlled studies in ET^37^ and PV^12^ did not identify a risk of LT related to HU. Likewise, interferon-alpha has not been clearly implicated with transformation.^31, 46, 47^ The associations of higher transformation risk related to combination or sequential therapy are not clear and may be representative of more aggressive disease requiring more therapy with ultimately higher risk of progression. It is not well understood what influence chronic therapy has on the acquisition of genetic aberrations related to MPN and their associated risk to transformation. Genetic mutations leading to drug resistance may also have a role in disease transformation whereby the presence of p53 mutations may have suspected involvement in this process.^48^

## Prognostic implications of the *JAK2V617F* mutation or its allele burden

Under normal circumstances, a polyclonal stem cell pool is responsible for hematopoiesis. In the setting of MPN, acquired mutations in hematopoietic stem cells lead to abnormal monoclonal or oligoclonal hematopoiesis. Additional genetic mutations may alter clinical phenotype pattern of each MPN, and during course of the disease, further genetic alterations can occur that promote disease progression and/or transformation.^48^ A major advance in understanding the pathogenesis of MPN was made with the discovery of the gain-of-function Janus kinase-2 (*JAK2V167F*) mutation in 2005.^49, 50, 51^ The most prevalent mutation in MPN is the *JAK2V617F* mutation on exon 14, which is present in 95% of PV and 50–60% of ET patients conferring a constitutive tyrosine kinase activity.^49, 50, 52^ Variable proportions of *JAK2V617F* mutant alleles are found in myeloid cell populations.^53^ The *JAK2V617F* mutation can be present in a heterozygous or a homozygous state, with the latter representing a mitotic recombination event resulting in uniparental disomy.^54^ Homozygosity is frequent in PV (25%) that contributes to their higher mutant allele burden.^54, 55^

A mutant allele dosage effect on clinical phenotype has been described in PV, whereby higher mutant allele burden correlates with higher severity of disease.^55, 56, 57^ Retrospective studies have identified that patients homozygous for *JAK2V617F* mutations are more likely to progress to post-PV MF.^56, 57, 58^ Rates of fibrotic transformation have been found to be 11.5% versus 1.4%^55^ and 23% versus 2%^59^ for homozygous compared with heterozygous *JAK2* mutants, respectively. In a study of 647 patients, 68 evolved to post-PV MF, with 78% at the time of evolution having mutant allele burdens of >50%.^21^ Similarly, Passamonti *et al.*^15^ identified 320 PV patients (median follow-up of 3.2 years) in whom a mutant allele burden ⩾50% was a significant risk factor for MF transformation. *JAK2V617F* allele burdens of ⩾50% have been associated with elevated white blood cell (*P=*0.034), thrombocytosis (*P=*0.010) and, accordingly, higher incidence of both thrombosis (*P=*0.032) and post-PV MF (*P=*0.018).^20^ Interestingly, in a study of 97 PV patients, 21% had MF transformation, and 22% developed leukemia whereby *JAK2V617F* allele burden (>50%) was associated with MF transformation (*P<*0.0001) but not leukemogenesis.^32^ Higher *JAK2V617F* allele burdens of >80% also correlated with advanced MF and greater splenomegaly.^60^ However, it should be noted that association of allelic burden and transformation, particularly risks of leukemic evolution, are not consistent in PV.^19, 61^

Among ET patients, 14.3% of homozygous patients for the *JAK2V617F* mutation developed MF as compared with 4.7% of heterozygous patients (*P=*0.011) or 1.6% of wild-type patients (*P=*0.001).^57^ In an evaluation of ET patients based on revised WHO criteria, Gangat *et al.*^37^ identified that the presence of *JAK2V617F* did not predict LT or inferior survival. In one of the largest ET studies comparing *JAK2V617F* mutated (*n=*414) to unmutated cases (*n=*362), there was no significant difference in survival, leukemic or MF transformation rates.^62^ Other studies confirmed the lack of correlation related to *JAK2* mutational status and disease transformation.^32, 63, 64^ Interestingly, in a large multicenter study by Barbui *et al.*^34^ the presence of *JAK2V617F* mutation significantly decreased the rate of MF progression (HR 0.37; 95% CI, 0.17–0.79, *P=*0.15) among ET patients but did not influence LT or overall survival. Finally, in comparing chronic phase to BP MPN, including both PV and ET, there was no statistical association between the time of LT or overall survival and *JAK2V617F* status. There is 20% less incidence of *JAK2V617F* mutations in BP MPN versus chronic phase MPN.^65^ In fact, it is not uncommon to find isolated acute leukemic clones to have reverted to a *JAK* wild-type status at the time of transformation.^66, 67^ Overall, *JAK2V617F* presence is not a prerequisite for LT and more likely additional genetic events are required in the setting of disease transformation.^65^

## Mutations influencing disease transformation

Following the discovery of *JAK2V617F* mutation, additional somatic mutations have been found and implicated in MPN pathogenesis, particularly in cases of *JAK2*-negative disease (Table 4). *MPL* is found at chromosome 1p34 and encodes the thrombopoietin receptor, which mediates signaling via the JAK–STAT (signal transducer and activator of transcription factor) pathway. *MPL*-acquired mutations (that is, *W515L*, *W515K*) occur in both ET and PMF, with approximately 1–4% in ET patients and mutually exclusive from *JAK2V617F*.^68, 69, 70^ Based on a population frequency of 6% in a MPN cohort study, *MPL* mutation status did not impact time to LT or overall survival.^65^ Additional studies have shown its lack of impact on post-ET MF or LT risk.^69, 70^ In a longer follow-up study, higher rates of post-ET MF, leukemia and lower overall survival rates occurred in *MPL*-mutated ET (3%, *n=*8) when compared with those with *JAK2* or *CALR* mutations.^71^ *CALR* mutations are rare in PV but occur in approximately 15–32%^71, 72, 73^ of ET cases, with higher incidences (49–71%) in *JAK2/MPL* unmutated ET.^72, 74, 75^ *CALR* is located on chromosome 19p13.2 and is a multi-functional Ca^2+^-binding protein chaperone.^76^ In a recent follow-up study of 299 ET patients over 12.7 years, *CALR* mutations versus *JAK2V617F* mutations were associated with younger age (*P=*0.002), male sex (*P=*0.01), higher platelet count (*P=*0.0004), lower hemoglobin (*P<*0.0001), lower leukocytosis (*P=*0.02) and lower thrombosis rates (*P=*0.04). Between the two mutational groups, LT and myelofibrotic transformation rates were not significantly different (*P=*0.28).^71^ In other studies, the *CALR* mutation has been associated with decreased or equal incidence of LT,^73, 77^ with no clear association with post-ET MF.^72, 73^ The role of *LNK* (*SH2B3*), an adaptor protein that is a negative regulator of thrombopoietin–*MPL*-mediated *JAK* activation pathway, is unclear. *LNK* mutations are associated with expansion of the myeloid progenitor precursors through the *JAK*–*STAT* pathway. In an analysis of 61 patients, *LNK* mutations were present in 9.8% of BP samples and were rare in chronic phase, suggesting that this mutation may be linked to leukemogenesis; however, they were not mutually exclusive of other MPN mutations and a direct genotype–phenotype correlation could not be concluded.^78^

Transcription factors have an important role in gene expression regulation and are often mutated in the setting of MPN. The gene encoding the Ikaros transcription factor (*IKZF1*) was shown in MPN to be the target of chromosome 7p deletions, with loss of *IKZF1* associated with LT.^79^ An important transcription factor that is responsible for cell cycle regulation and DNA damage response is p53 (encoded by *TP53* gene), a tumor-suppressor protein that has been implicated in LT in MPN.^80, 81, 82^ Among 22 patients with post-MPN AML, 45.5% had a form of *p53* mutation.^82^ In analyzing post-MPN AML, the presence of p53 mutations was an adverse prognostic factor for overall survival (HR 2.67; 95% CI, *P=*0.006).^83^ Likewise, the *RUNX1/AML1* gene encodes a transcription factor involved in hematopoiesis, and mutations are linked to LT in MPNs.^51, 80, 84^ Finally, splicing factors have also been implicated with recurrent mutations in *SRSF2*, *ZRSF2*, *U2AF1* and *SF3B1* identified among 22 MPN patients with leukemia.^85^

In MPN-BP, genomic alterations are threefold (*P<*0.001) more abundant compared with chronic phase.^65^ The spectrum of mutations in MPN-derived leukemia differs from *de novo* AML where in the latter we commonly see mutations in *FLT3*, *NPM1* and *DNMT3a*.^83, 86, 87^ In post-MPN AML, gene sequencing studies have identified frequent mutations in epigenetic regulators such as ten-eleven translocation 2 (*TET2*), additional sex combs-like (*ASXL1*) and isocitrate dehydrogenase (*IDH1/2*) at the time of LT.^81, 85, 88, 89^ Rampal *et al.*^81^ identified additional mutations using high-throughput sequence analysis on post-MPN AML patients, including: *CALR*, *MYC*, *PTPN11* and *SETBP1*. In *JAK2V617F*-mutated subgroups, common co-occurring mutations were *TP53* (44%), *ASXL1* (44%) and *IDH2* (44%), whereas *CALR* (43%), *ASXL1* (38%) and *SRSF2* tended to occur in JAK2 wild type post-MPN. TET enzymes catalyze conversion of 5-methylcytosine to 5-hydroxymethycytosine, which leads to DNA methylation. The frequency of *TET2* mutations are 5% in ET and 16% in PV patients.^90^ The incidence of *TET2* mutations in MPN-BP has been reported as 17%, but the presence of the mutation among PV and PMF patients did not seem to affect LT or survival rates.^90^ The enzymes *IDH1/2* catalyze the conversion of isocitrate to alpha-ketoglutarate, which acts as a co-factor for *TET2* whereby intact IDH activity is important for protection against oxidative stress.^51^ Mutations of *IDH1* and *IDH2* cause inhibition of *TET2* activity, lead to decreased DNA methylation and impaired hematopoietic differentiation.^91^ There is a low incidence of *IDH1/2* mutations in PV (1.9%) and ET (0.8%); however, in MPN-BP, *IDH* mutations are as high as 21.6%, suggesting an association with LT (*P<*0.01) and confer a worse survival (*P=*0.01).^92^ Finally, *ASXL1* is a nuclear polycomb protein with mutations found on chromosome 20q1.1 that affect the regulation of transcription and RAR-mediated signaling. *ASXL1* mutations are rare in ET and PV but are most frequently found in post-ET MF and may be related to its pathogenesis.^93^

## Cytogenetic risk factors

It is anticipated that our expanding knowledge of the genetic profiles of MPN will have an important role in predicting disease outcomes. Tefferi *et al.*^3^ showed for the first time the prognostic relevance of karyotype in an international PV study. Similarly, Dingli *et al.*^94^ illustrated that cytogenetic findings likely superseded disease-related characteristics such as age and anemia for predicting survival among PV and ET patients with secondary MF. Frequently reported karyotype abnormalities in MPN-BP are complex often involving chromosomes 1, 9, 5 and 7 abnormalities.^8, 19, 84, 95, 96^ In general, cytogenetic abnormalities are estimated in 15% of PV patients.^19, 61^ Among transformed PV patients, with no prior treatment exposures aside from phlebotomy, the most frequent cytogenetic abnormalities included: +1q, +8, +9, and 20q−.^96^ In ET, most patients have a normal karyotype at the time of diagnosis, with an overall prevalence of cytogenetic anomalies being <10%.^97, 98, 99^ Despite having abnormal cytogenetics at the time of diagnosis, ET patients do not have shorter survival or higher risks of myelofibrotic transformation or LT.^97^ At the time of LT, most patients have a detectable abnormality.^97, 98, 100^ Overall, single-nucleotide polymorphism arrays have identified changes of chromosomes 1q, 7q, 5q, 6p, 7p, 16q, 19q, 21q, 22q and 3q associated with post-MPN AML.^65, 84^ High-resolution single-nucleotide polymorphism arrays have identified established targets relating to disease progression that include: *MYC* (chromosome 8), *ETV6* (chromosome 12), *TP53* (chromosome 17), and *RUNX1* (chromosome 21).^65, 84^ Clearly, in other myeloid malignancies and in primary MF, cytogenetics has an important role in prognosis, and currently our knowledge of its implication in PV and ET is expanding. Ultimately, with new technologic advancements, identifying candidate genes involved in transformation of both PV and ET into MF and AML/MDS can provide insight into the complex pathogenesis of MPN and assist in the development of therapeutic targets for prevention of transformation.

## Conclusion

Both PV and ET are *BCR-ABL1*-negative MPN with increased morbidity and mortality mainly attributed to thrombohemorrhagic complications with progression to AML and MF leading to a dismal prognosis, particularly in the setting of LT where median survival is significantly shortened to <6 months. Allogeneic stem cell transplantation remains the only curative option in few eligible patients and new therapeutic agents remain in development. The underlying mechanism for transformation to either MF or BP remains unclear and is likely multifactorial. There is a need to improve the identification of high-risk patients. Understanding the genetic mutations that lead to disease progression and transformation is a current research focus, and with advancements in genetic profiling, the pathogenesis of MPN will become even more complex. It is anticipated that establishing genetic profiles within MPN will allow better classification of patients to improve clinical management and treatment.

## Figures and Tables

**Table 1:** Predicting long-term outcomes for patients with polycythemia vera and essential thrombocythemia

	*PV*	*ET*
Overall survival (years)	13.5–24	11–22.6
		
Leukemic transformation (median time in years)	4.6–19	6.3–14.5
Cumulative risks	—	0.2–0.3% at 5 years
	2.3–14.4% at 10 years	0.7–3% at 10 years
	5.5–18.7% at 15 years	2.1–5.3% at 15 years
	7.9–17% at 20 years	8.1% at 20 years
		
Myelofibrosis transformation (median time in years)	8.5–20	7.3–16
Cumulative risks	—	0.1–1% at 5 years
	4.9–6% at 10 years	0.8–4.9% at 10 years
	6–14% at 15 years	4–11% at 15 years
	26% at 20 years	19.9% at 20 years
		
Risk algorithms	IWG-MRT^3^—to predict overall survival	IPSET^4^—to predict survival and occurrence of thrombosis

*Risk factors*		
Age	⩾67 (5 pts) vs 57–66 (2 pts)	⩾60 (2 pts)
WBC	⩾15 × 10^9^/l (1 pt)	⩾11 × 10^9^/l (1 pt)
Thrombosis	Venous thrombosis (1 pt)	Yes (1 pt)

*Category*		
Low risk	0 pts	0 pts
Intermediate risk	1–2 pts	1–2 pts
High risk	⩾3 pts	3–4 pts

*Survival (years)*		
Low risk	26	NR
Intermediate risk	15	24.5
High risk	8.3	13.8

Abbreviations: ET, essential thrombocythemia; IWG-MRT, International Working Group for MPN Research and Treatment; IPSET, International Prognostic Score for essential thrombocythemia; NR, not reported; pt/pts, patient/patients; PV, polycythemia vera; WBC, white blood cell.

**Table 2:** Studies evaluating outcomes in polycythemia vera

	*Kiladjian* et al.^7^	*Passamonti* et al.^11^	*Finazzi* et al.^12^	*Marchioli* et al.^13^	*Gangat* et al.^14^	*Passamonti* et al.^15^	*Abdulkarim* et al.^16^	*Tefferi* et al.^3^	*Bonicelli* et al.^17^	*Stein* et al.^18^	*Sever* et al.^19^	*Tefferi* et al.^2^	*Bai* et al.^20^
*N*	164	396	1638	1638	459	320	150	1545	327	204	133	267 (Mayo) 310 (Italy)	272
Median F/U (years)	11.4	9.6	2.8	2.7	5.3	3.2	15	6.9	11	8 (age ⩽45 years) 4.5 (age ⩾65 years)	7.5	11.8 11.1	6

*MF*													
*N*	14 (8.5%)	5.1 (3.3–7.8)^a^	NR	38 (2.3%)	54 (12%)	8 (2.5%)	13 (8.5%)	138 (9%)	37 (11%)	26 (12.7%)	11 (8%)	34 (12.7%) 65 (21%)	63 (23%)^b^
Median time to MF from Dx (years):	12.5	13	NR	NR	10.5	NR	NR	NR	9.6	20 (age ⩽45 years) 8 (age ⩾65 years)	8.5^c^	9.6	NR
Risk factors	None	Sequential use of ⩾2 myelosuppressive agents compared with Pipobroman	NR	Disease duration >10 years	Age ⩾60 years	*JAK2V617F* allele burden ⩾50%	Splenomegaly Reticulin grading	NR	None	Median age at MF Dx	None	None	Splenomegaly Plt>550 × 10^9^/l *JAK2V617F* allele burden ⩾50%

*Leukemia*													
*N*	32 (19.5%)	5.3 (3.5-8)^a^	22 (1.3%)	21 (1.3%)	34 (7.4%)	10 (3%)	13 (8.5%)	50 (3%)	30 (9.2%)	7 (3%)	4 (3%)	18 (6.7%) 10 (3.2%)	6.6 (3.7-10.8)^d^
Median time to LT from Dx (years):	9.6	14	8.4	NR	10.5	NR	NR	10.8	4.6	19 (age ⩽45 years) 7 (age ⩾65 years)	8.5^c^	NR	NR
Risk factors	WBC⩾10 × 10^9^/l	Sequential use of ⩾2 myelosuppressive agents compared with HU or Pipobroman	Age ⩾70 years P32 Busulphan Pipobroman Cytoreductive drug alone or in combo Low cholesterol (⩽150 mg/dl)	Age ⩾70 years Cytoreductive agents other than HU or IFN	WBC⩾10 × 10^9^/l WBC⩾15 × 10^9^/l	None	Splenomegaly Reticulin grading	Age >61 years WBC ⩾15 × 10^9^/l Abnormal karyotype P32/CMB alone Pipobroman	Female	None	None	None	Plt<100 × 10^9^/l
													
Median Survival (years):	15.5	20	NR	NR^e^	22.7	NR	NR^f^	18.9^g^	17.5 (age <65 years) 6.4 (age ⩾65 years)^h^	NR	24	13.5^i^	NR^j^
Risk factors	Age ⩾60 years WBC⩾10 × 10^9^/l	Thrombosis	NR	Age >65 years Thrombosis	Age ⩾60 years WBC⩾15 × 10^9^/l Arterial thrombosis	Age ⩾60 years	Splenomegaly	Age >61 years WBC⩾15 × 10^9^/l Thrombosis Abnormal karyotype Pruritus^k^	Age >70 years WBC>13 × 10^9^/l Thrombosis	NR	None	Age <60 years	Age >65 years WBC>25 × 10^9^/l Thrombosis

Abbreviations: CMB, chlorambucil; Dx, diagnosis; HU, hydroxyurea; IFN, interferon; LT, leukemic transformation; MF, myelofibrosis; NR, not reported; WBC, white blood cell.

a Incidence per 1000 person-years (95% CI).

b Incidence of 31 (95% CI=24.12–40.16) per 1000 person/year.

c Median time to leukemic or myelofibrotic transformation.

d Incidence rate per 1000 person/year with 19% leukemic transformation from post-PV MF population (*n=*63).

e Overall mortality rate of 3.7 per 100 persons per year.

f Median overall survival 40% at 10 years.

g Median overall survival 14.1 years based on most mature follow-up cohort (*n=*337).

h Median overall survival 72% at 10 years.

i Based on Mayo cohort.

j 83% median overall survival at 10 years.

k Favorable risk factor.

**Table 3:** Studies evaluating outcomes in essential thrombocythemia

	*Passamonti* et al.^11^	*Chim* et al*.*^29^	*Wolanskyj* et al.^28^	*Gangat* et al.^37^	*Passamonti* et al.^30^	*Palandri* et al.^31^	*Girodon* et al.^35^	*Abdulkarim* et al.^16^	*Barbui* et al.^34^	*Malak* et al.^32^	*Tefferi* et al.^2^
*N*	435	231	322	605	605	386	311	130	891	105	292 (Mayo) 284 (Italy)
Median F/U (years)	9.3	NR	13.6	7	5.6	9.5	9.5	15	6.2	7.5	17.3 10.7

*MF*											
*N*	1.6 (0.8–3.4)^a^	7 (3%)	NR^b^	NR	17 (2.8%)	20 (5%)	NR	7 (5%)	32 (4%)	12 (13%)	29 (9.9%) 26 (9.2%)
Median time to MF from Dx (years)	10.9	8	12.4	NR	9.1	7.25	NR	NR	NR	16	NR
Risk factors	NR	NR	None	NR	Anemia^c^	Sequential use of cytotoxic drugs	NR	Anemia Leukocytosis Reticulin BM cellularity	BM histology Age >60 years Hgb<120 g/l Absent *JAK2V617F*	None	*MPL* mutated

*Leukemia*											
*N*	1.2 (0.5–2.8)^a^	4 (1.7%)	NR^d^	20 (3.3%)	14 (2.3%)	6 (1.5%)	18 (5.8%)	11 (8%)	8 (1%)	7 (10%)	12 (4.1%) 4 (1.4%)
Median time to LT from Dx (years)	14.5	10	13.8	11.5	11	8.5	6.3	NR	NR	7.4	NR
Risk factors	None	Previous MF Melphalan WBC>11 × 10^9^/l	None	Anemia^e^ Plts ⩾1000 × 10^9^/l Age (continuous variable)	Age >60 years	Sequential use of cytotoxic drugs	None	Anemia Leukocytosis Reticulin BM cellularity	BM histology Thrombosis Plts>1000 × 10^9^/l	None	None
											
Median survival (years)	22.6	13	18.9	18	22.3	NR	11	NR^f^	14.7	NR^g^	19.8^h^
Risk factors	Thrombosis Male	Age >60 years	Age ⩾60 years WBC⩾15 × 10^9^/l Tobacco use DM	Age ⩾60 years WBC⩾15 × 10^9^/l Anemia^e^ Thrombosis DM Smoking	Age >60 years Thrombosis	Age ⩾60 years WBC⩾15 × 10^9^/l HTN DM Thrombosis	Age >60 years WBC>11 × 10^9^/l Anemia	Anemia	Age >60 years WBC>11 × 10^9^/l Hgb <120 g/l Thrombosis BM histology	NR	Age ⩾60 years

Abbreviations: BM, bone marrow; DM, diabetes mellitus; Dx, diagnosis; HTN, hypertension; HU, hydroxyurea; LT, leukemic transformation; MF, myelofibrosis; NR, not reported; Plts, platelets; WBC, white blood cell.

a Incidence per 1000 person-years (95% CI).

b 3.8% cumulative probability of MF transformation at 10 years (95% CI=1.4–6.1).

c Hgb<125 g/l in females, <135 g/l in males.

d 1.4% 10-year cumulative probability of AML transformation (95% CI=0–3).

e Hgb<120 g/l in females, <135 g/l in males.

f Overall survival 58% at 10 years.

g Overall survival 83% at 10 years.

h Based on Mayo cohort.

**Table 4:** Intrinsic risk factors for disease transformation in PV and ET

*Transformation*	*Clinical risk factors*	*Genetic risk factors*
Post-PV MF	Age	*JAK2V617F* allele burden
	Leukocytosis	
	Disease duration	
	Reticulin fibrosis	
	Splenomegaly	
Post-PV Leukemia	Age	Abnormal karyotype
	Leukocytosis	*TP53*
	Reticulin fibrosis	*RUNX1*
	Splenomegaly	
Post-ET MF	Age	Absent *JAK2V617F* mutation
	Leukocytosis	*ASXL1*
	Anemia	
	Reticulin fibrosis	
Post-ET leukemia	Age	*TP53*
	Leukocytosis	*RUNX1*
	Anemia	
	Reticulin fibrosis	
	Thrombosis	
	Platelets ⩾1000 × 10^9^/l	

Abbreviations: ET, essential thrombocythemia; MF, myelofibrosis; PV, polycythemia vera.
